# Does Food Neophobia Affect Dietary Diversity and Treatment Adherence in Individuals With Type 2 Diabetes Mellitus? A Pilot Study From Türkiye

**DOI:** 10.1002/fsn3.72036

**Published:** 2026-06-21

**Authors:** Merve Sena Topkaya, Dilara Berşan Konyalıgil, Berna Betül Cihan, Kübra Tel Adıgüzel, Ertuğrul Ekici, Nevra Koç

**Affiliations:** ^1^ Department of Nutrition and Dietetics, Gülhane Health Sciences Institute University of Health Sciences Turkey Ankara Turkey; ^2^ Department of Nutrition and Dietetics, Faculty of Health Sciences Atılım University Ankara Turkey; ^3^ Department of Nutrition and Dietetics Çankırı State Hospital Çankırı Turkey; ^4^ Department of Nutrition and Dietetics, Gülhane Health Sciences Faculty University of Health Sciences Turkey Ankara Turkey; ^5^ Department of Internal Medicine Çankırı State Hospital Çankırı Turkey

## Abstract

Food neophobia is defined as reluctance to eat new foods and avoidance of unfamiliar products. In individuals diagnosed with type 2 diabetes mellitus (T2DM) may be associated with reduced dietary diversity and poorer adherence to T2DM treatment. Therefore, this study aimed to examine the association between food neophobia, dietary diversity, and adherence to treatment in individuals with T2DM. The cross‐sectional research included 35 individuals with T2DM who were monitored at the Internal Medicine Outpatient Clinic of Çankırı State Hospital, and 35 healthy individuals the same outpatient clinic. Demographic characteristics, Adherence Scale to Type 2 Diabetes Mellitus Treatment, Dietary Diversity Score and the Foods Neophobia Scale were recorded using a questionnaire administered by the researchers via face‐to‐face interviews. HbA1c levels were obtained from the hospital's electronic database. The findings indicated that elevated food neophobia scores were linked to lower dietary diversity and poorer treatment adherence among individuals with T2DM. However, no statistically significant difference was found between the healthy participants and those with T2DM regarding food neophobia scores. These findings suggest that food neophobia may be related to lower dietary diversity and poorer treatment adherence in individuals with T2DM. Addressing food neophobia may be an important strategy to increase dietary diversity and improve treatment adherence in individuals with T2DM.

## Graphical abstract

This single‐center study compared 35 individuals with type 2 diabetes mellitus and 35 healthy controls. Demographic characteristics, treatment adherence, dietary diversity, and food neophobia were assessed using validated scales. Food neophobia in individuals with type 2 diabetes was associated with lower dietary diversity and poorer treatment adherence. However, no significant difference in food neophobia scores was observed between participants with type 2 diabetes and healthy controls.

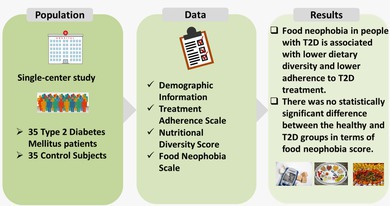

## 1 Introduction

Diabetes mellitus (DM) is a metabolic disorder characterized by hyperglycemia resulting from impaired insulin secretion, insulin action or both (American Diabetes Association 2014). According to the 10th edition of the International Diabetes Federation Diabetes Atlas, the global number of adults aged 20–79 years living with diabetes is projected to increase from 536.6 million (10.5%) globally in 2021 to 783.2 million (12.2%) by 2045. According to the same report, in Türkiye, this number was estimated to increase from 9 million to 13.4 million over the same period (Sun et al. 2022).

Disease management requires ongoing self‐care throughout the patient's lifetime, including a diabetes‐specific diet, regular physical activity, blood glucose monitoring, and compliance with medication regimens (Pourhabibi et al. 2022). Diet is as important as medical intervention in the management of diabetes mellitus. Ensuring glycemic control plays an important role in the therapeutic strategy to prevent microvascular and macrovascular complications. Factors such as diet lists to be followed to control the disease, prohibited foods and the fact that it is a chronic disease cause anxiety in patients and cause the mind to be preoccupied with food and weight control. For these reasons, deterioration in patients' eating attitudes and behaviors can be observed (Kaynarpunar and Akman 2021).

Food neophobia has been defined as an unwillingness or avoidance to eat new foods. It is a personality trait that affects people's daily food choices (Tian and Chen 2021). Numerous studies have reported that food neophobia is inversely related to the intake of vegetables, fruits, and fish (Jezewska‐Zychowicz et al. 2021; Karaağaç and Bellikci‐Koyu 2023; Sarin et al. 2019). A lack of motivation to try new foods limits the variety of foods consumed, which is essential for a balanced diet. This limited variety may make it difficult to follow the dietary guidelines needed for optimal diabetes management (Mameli et al. 2019).

Food neophobia and food selectivity can potentially affect glycemic control by making adherence to overall diabetes management more difficult (Quick et al. 2014); however, no research has examined these relationships in patients with T2DM. In this context, we impact the effect of food neophobia on treatment adherence and dietary diversity in patients with T2DM. It was hypothesized that higher levels of food neophobia would be associated with lower dietary diversity and adherence to dietary recommendations.

## 2 Materials and Methods

This cross‐sectional study included 35 individuals with T2DM mellitus who were followed up at the Internal Medicine Polyclinic of Çankırı State Hospital between June and August 2024, and 35 healthy individuals attending the same polyclinic. Patients who had been diagnosed with diabetes for less than 1 year, those with any chronic disease other than T2DM, and those with eating disorders were excluded from the study.

The study was approved by the Research Ethics Committee of Gülhane Health Sciences University on 28.05.2024 with the number 2024‐245. The informed voluntary consent form was read and signed by the subjects included in the study.

According to the two‐tailed independent samples *t*‐test analysis at the 0.05 significance level, with 0.95 power and an effect size of *d* = 0.5422, and based on the study by Eşer et al. (2018), it was determined that a total of at least 64 individuals were required, including at least 32 individuals with T2DM and 32 healthy individuals.

### 2.1 Data Collection

Demographic characteristics, the Patient Compliance with Treatment for T2DM Scale, the Dietary diversity score, and the Foods Neophobia Scale were collected using a questionnaire administered by the researchers during face‐to‐face interviews. All questionnaires were administered face‐to‐face and validated during data collection. Therefore, the study had no missing data, all analyses were performed using a complete case study approach, and no data completion was carried out. HbA1c levels were obtained from the hospital electronic database. Data collection and measurement methods were applied identically to both the T2DM group and the healthy control group to ensure comparability.

### 2.2 Adherence Scale to Type 2 Diabetes Mellitus Treatment

The scale was developed by Demirtaş, and validity and reliability studies have been conducted. It is designed to be administered to individuals diagnosed with diabetes for at least 1 year (Demirtaş 2014) The 30‐item, five‐point Likert scale is arranged from “totally agree” (1 point) to “totally disagree” (5 points), with each option increasing by 1 point.

The scale includes 13 positive and 17 negative statements. Questions 2, 4, 6, 7, 9, 10, 11, 12, 14, 18, 20, 21, 22, 24, 27, 28, and 30 are reverse‐coded. The highest possible score on the scale is 150, and a lower score indicates high patient adherence to Type II DM treatment.

Evaluation is based on total scores. Accordingly; A total score in the 0%–20% range (30–54 points) indicates good treatment adherence, a 20%–80% range (55–125 points) indicates moderate adherence, and a 80%–100% range (126–150 points) indicates poor treatment adherence.

The scale consists of seven sub‐dimensions. The first factor comprises items 11, 12, 14, 21, 23, 24, 25, 29, and 30 and is called “attitude and emotional factors.” The second factor consists of items 3, 7, 8, 9, 13, 16, and 27 and is expressed as “knowledge and personal factors.” The third factor includes items 5, 20, and 28 and is named “lifestyle change.” The fourth factor consists of items 10, 18, and 22 and is defined as “feelings of anger.” The fifth factor, comprising items 1, 15, 17, and 26, is called “Feelings and behaviors appropriate to adherence.” The sixth factor consists of items 6 and 31 and is expressed as “Talks on diet.” The seventh factor includes items 2 and 4 and is named “denial.”

Low scores on the sub‐dimensions indicate that the individual is exhibiting the expected positive attitudes and behaviors during the treatment process. Conversely, high scores indicate low adherence to treatment and an inability to adequately demonstrate the expected emotions and behaviors (Demirtaş 2014).

### 2.3 Dietary Diversity Score

The Dietary Diversity Score (DDS) table consists of five food groups (grains, vegetables, fruits, meat, and dairy) and 23 subgroups. Each main food group is evaluated on a scale of 2 points, with a maximum total score of 10 (Kant et al. 1993). Food consumption status was assessed based on 2‐day food consumption records covering weekdays and weekends. For a food to be considered consumed, at least half of the designated portion size must have been consumed. Portion sizes were determined using the USDA (United States Department of Agriculture) Food Pyramid and the Turkey Specific Nutrition Guide (Tüber 2022).

Each main group is worth a maximum of 2 points. The score of a main group is obtained by dividing the total number of subgroups under that group by the number of subgroups consumed (Group Score = (2/n) × number of subgroups consumed) (Table 1).

**TABLE 1:** Calculation of the dietary diversity score.

Main food group	Sub‐food groups	Number of subgroups	Scoring method
Cereals	White bread; biscuits/cornbread; pasta (noodles, vermicelli, lasagna, etc.); whole‐grain bread/cereals (bulgur, etc.); breakfast cereals/popcorn; rice; refined flour and grains	7	(number of consumed subgroups/7) × 2
Vegetables	Salad vegetables (lettuce, broccoli, etc.) and cooked vegetables in foods (spinach, etc.); potatoes; tomatoes and tomato products; starchy foods; dried legumes; yellow vegetables (carrots, zucchini); green vegetables (mint, basil, radish)	7	(number of consumed subgroups/7) × 2
Fruits	Fruits and fruit juices; citrus fruits/strawberries/blackberries/raspberries/melon/watermelon	2	(number of consumed subgroups/2) × 2
Meat	Red meat; white meat; fish; eggs	4	(number of consumed subgroups/4) × 2
Dairy	Milk/buttermilk/kefir; yogurt; cheese	3	(number of consumed subgroups/3) × 2
Total Score			Maximum: 10 points

Carbonated and alcoholic beverages, coffee, sugar and sugar products, fatty products, and bakery products were not included in the dietary diversity score calculation. The DDS was classified as low variety (DDS < 3.5), medium variety (3.5 ≤ DDS ≤ 6.5) and variety (DDS > 6.5) (Tüber 2022).

### 2.4 Food Neophobia Scale

In our study, the 10‐item Food Neophobia Scale was used. Turkish validity and reliability of the scale was established by Uçar, and the Cronbach alpha value was found to be 0.805 (Uçar et al. 2021). The scale, which is a seven‐point Likert type, is rated from “strongly disagree” (1 point) to “strongly agree” (7 points), with a total scores range from 10 to 70. Items 1, 4, 6, 9, and 10 in the scale were reverse‐scored. Higher scores reflect greater levels of food neophobia, whereas lower scores indicate a more positive attitude toward trying new foods.

In the study, individuals were divided into three groups. The scoring was based on the mean (𝑥) and standard deviation (S) of the total scores. Individuals with a Food Neophobia Scale score of <𝑥 ±1S were classified as neophilic, 𝑥±1S as neutral, and >𝑥±1S as neophobic with high fear of novel foods (Uçar et al. 2021).

### 2.5 Statistical Analyses

The data collected from the questionnaires and measurements were statistically analyzed using IBM SPSS Statistics version 21.0. The mean, standard deviation, minimum, and maximum values were calculated for the collected data. Continuous variables are expressed as mean ± standard deviation (X̄±SS), while categorical variables are presented as number (*n*) and percentage (%). When comparing two independent groups with normally distributed data, the independent samples *t*‐test was used; for comparisons involving more than two groups, one‐way ANOVA was applied. The significance of the distribution between groups in descriptive statistics was analyzed using Fisher's chi‐square test. Multiple regression analysis was conducted to examine the relationship among variables. A *p* value below 0.05 was considered statistically significant.

## 3 Results

A total of 70 participants were included in the study; comprising 35 individuals with type 2 diabetes, and 35 healthy control. Table 2 shows the distribution of age, gender, height, weight, BMI, and education level of the subjects. The mean age of the control group was 62.1 ± 18.49 years, and the mean age of the subjects with T2DM was 62.7 ± 13.09 years. A total of 70 people were included in the study; 57.1% of the control group were female and 42.9% were male; 71.4% of the individuals with T2DM were female and 28.6% were male.

**TABLE 2:** Sociodemographic characteristics.

Variable	Control Group	T2DM Group
Age (years) (X̄±SD)	62.1 ± 18.49	62.7 ± 13.09
Gender s (%)		
Female	20 (%57.1)	25 (%71.4)
Male	15 (%42.9)	10 (%28.6)
Weight (kg) (X̄±SD)	75.3 ± 16.74	75.8 ± 12.32
Height (m) (X̄±SD)	163.0 ± 7.22	159.0 ± 7.46
BMI (kg/m^2^) s (%)		
Normal weight	14 (%40.0)	6 (%17.1)
Over weight	11 (%31.4)	10 (%28.6)
Obese (Class I)	5 (%14.3)	13 (%37.1)
Obese (Class II)	1 (%2.9)	6 (%17.1)
Obese (Class III)	4 (%11.4)	0 (%0)
Education level s (%)		
Illiterate	7 (%20.0)	5 (%14.3)
Literate‐primary school graduate	20 (%57.1)	26 (%74.3)
Middle school graduate	4 (%11.4)	2 (%5.7)
High school graduate	3 (%8.6)	2 (%5.7)
University graduate or higher	1 (%2.9)	0 (%0)

Table 3 shows the assessment of the characteristics of the individuals with T2DM in relation to their disease status. Mean duration of diagnosis was 13.1 ± 7.89 years and the HbA1C level was 9.3 ± 2.66. While 31.4% of individuals received diabetes care education from a nurse, 57.1% received dietary education from a dietitian.

**TABLE 3:** Characteristics of the disease.

Variable	s (%)
Duration of diagnosis (X̄±SD)	13.1 ± 7.89
Applied treatment	
Diet	1 (%2.9)
Oral Anti Diabetic	17 (%48.6)
Insulin	10 (%28.6)
Insulin + Oral Anti Diabetic	7 (%20.0)
Control frequency	
Once a month	1 (%2.9)
Once every 2–3 months	13 (%37.1)
1 time in 6 months	3 (%8.6)
1 time per year	2 (%5.7)
Less than once a year (infrequently)	16 (%45.7)
Receiving diabetes care education from a nurse	
Received	11 (%31.4)
Not received	24 (%68.6)
Receiving dietary education from a dietitian	
Received	20 (%57.1)
Not received	15 (%42.9)
Complication status	
Diabetic retinopathy	3 (%8.5)
Diabetic nephropathy	1 (%2.8)
Hypertension	3 (%8.5)
Previous heart attack (MI)	3 (%8.5)
Metabolic control indicators and lifestyle characteristics	
HbA1C (X̄±SD)	9.3 ± 2.66
Alcohol use	
Present	1 (%2.9)
Absent	34 (%97.1)
Cigarette	
Smoker	4 (%11.4)
Nonsmoker	31 (%88.6)

When evaluating the DDS and FNS status of the individuals included in the study, no statistically significant differences were found between the scores of control group (6.0 ± 1.28, 41.8 ± 11.38, respectively) and those of the individuals with T2DM (6.0 ± 1.82, 45.6 ± 13.50, respectively). The adherence to treatment score for the individuals with T2DM was 85.2 ± 22.69 (Table 4).

**TABLE 4:** Assessment of dietary diversity, food neophobia, adherence to type 2 diabetes mellitus treatment scores of study participants.

Variable	Control Group	T2DM Group	*p*
Dietary diversity score	6.0 ± 1.28	6.0 ± 1.82	0.990
Food neophobia score	41.8 ± 11.38	45.6 ± 13.50	0.198
Adherence to treatment score	—	85.2 ± 22.69	—

*Note:* The Independent Samples *t*‐test, *p* < 0.05.

When the DDS and FNS status of the study participants were evaluated using the Fisher Freeman–Halton Exact Test, a significant difference between the groups was found for both groups (*p* < 0.001). While 77.8% of neophilic individuals with T2DM and 68.8% of neutral individuals with T2DM had a varied DDS, there were no individuals with a less varied DDS in either group. While 70.0% of neophilic individuals had a less varied DDS, only 10.0% had a varied DDS. When we analyzed the control group, 25.0% of neophilic individuals and 43.5% of neutral individuals had a varied DDS, while there were no individuals with a less varied DDS in either group. In neophobic individuals, all were found to have a less diverse DDS.

When analyzing Table 5, it was observed that the DDS of individuals with T2DM differed significantly according to their food neophobia status (*F* = 17.006) (*p* < 0.001). The Welch ANOVA test was used to determine between which food neophobia states the dietary diversity scores of the individuals with T2DM differed. According to the test results, it was observed that the dietary diversity score of individuals with food neophobia (X̄ = 4.0) was significantly lower than that of individuals who were neophilic (X̄ = 7.0) and neutral (X̄ = 6.7) (*p* < 0.05). Bulleted lists look like this:

**TABLE 5:** ANOVA results for dietary diversity score according to food neophobia in individuals with type 2 diabetes mellitus.

Variable	Groups	*N*	X̄±SD	*F*	Df	*p*	Difference
Dietary diversity score	Neophilic^1^	9	7.0 ± 1.58		2		3–1,2
	Neutral^2^	16	6.7 ± 0.72	17.006	13.542	0.001*	
	Neophobic^3^	10	4.0 ± 1.72				

*Note:* The dietary diversity score was significantly different among the groups (*p* = 0.001), with the neophobic group (Group 3) having significantly lower scores than the neophilic (Group 1) and neutral (Group 2) groups; The variance of groups was not homogeneously distributed, Welch test is considered, *p < 0.001.

When analyzing Table 6, it was observed that the T2DM treatment adherence scores of the individuals with T2DM showed a significant difference according to their food neophobia status (*F* = 16,065) (*p* < 0.05). The Welch ANOVA test was used to determine between which food neophobia states the adherence scores of individuals with T2DM differed. According to the test results, it was observed that the treatment adherence scale score of individuals with food neophobia (X̄ = 111.8) was significantly higher than that of neophilic (X̄ = 59.5) and neutral (X̄ = 83.0) individuals. It was also observed that the treatment adherence scale score of neutral individuals was significantly higher than that of neophilic individuals. A high score indicates that the individual is not compliant with the treatment and does not show the expected emotions and behaviors.

**TABLE 6:** ANOVA results on adherence to treatment according to food neophobia in individuals with type 2 diabetes mellitus.

Variable	Groups	*N*	x̄±SD	*F*	df	*p*	Difference
Adherence to treatment scale	Neophilic^1^	9	59.5 ± 22.20		2		2 > 1 3 > 1,2
	Neutral^2^	16	83.0 ± 7.86	16.065	12.516	0.004*	
	Neophobic^3^	10	111.8 ± 30.07				

*Note:* Adherence to treatment was significantly different among the groups (*p* = 0.004). The neutral group had significantly higher scores than the neophilic group (2 > 1), while the neophobic group had significantly higher scores than both the neophilic and neutral groups (3 > 1, 2); Variance of groups was not homogeneously distributed, Welch test is considered, *p < 0.05.

In Table 7, the associations of food neophobia score with dietary diversity score and adherence to treatment in the individuals with T2DM score among individuals with T2DM were evaluated by multiple regression analysis. A statistically significant positive moderate relationship was found between the food phobia score and treatment adherence scale scores, while a statistically significant negative moderate relationship was identified between the food phobia scores and the dietary diversity scores (*p* < 0.05). Since a higher adherence to treatment scale score indicates poorer adherence, these findings suggest that higher food neophobia scores were related to poorer treatment adherence and lower dietary diversity.

**TABLE 7:** Results of multiple regression analysis of the relationship between dietary diversity score and adherence according to food neophobia in individuals with type 2 diabetes mellitus.

Variable	B	*p*	T	95% confidence interval
Dietary diversity score	−2.453	0.014*	−2.597	−4.377: −0.529
Adherence to treatment score	0.270	0.000*	4.343	0.143: 0.396

* *p* < 0.05.

## 4 Discussion

Consistent with the study hypotheses, the findings of this study indicate that, among individuals with T2DM, higher food neophobia scores were associated with lower dietary diversity and poorer adherence to T2DM treatment. However, food neophobia scores were not statistically different between healthy individuals and individuals with T2DM.

In this study, the food neophobia score was 41.8 ± 11.38 in healthy individuals and 45.6 ± 13.50 in individuals with T2DM. There was no statistically significant difference between the healthy and T2DM groups in terms of the food neophobia score (*p* > 0.05) (Table 4). The fact that there was no difference between individuals with T2DM and healthy individuals in terms of food neophobia score may be due to the fact that this trait is a common tendency in the general population. The similar distribution of food neophobia scores among individuals regardless of health status suggests that food neophobia may be related not only to disease status but also to a wider range of individual and environmental factors.

In a study conducted among patients with T2DM in Türkiye, the mean treatment adherence score of the patients was found to be 74.22 ± 12.13 (Eker 2021). In a study conducted by Arı and Özdelikara (2022), the mean adherence score of patients with T2DM was 82.77 ± 9.19. In our study, the adherence score of individuals with T2DM was 85.2 ± 22.69 (Table 4). Our findings were consistent with previous studies, as treatment adherence among individuals with T2DM was moderate in all three studies.

Studies have associated food neophobia with adverse health outcomes such as poorer diet quality and increased risk of T2DM (Sarin et al. 2019; Knaapila et al. 2015). For example, in a study examining food neophobia in adult populations in Finland and Estonia, higher food neophobia scores were linked to poorer diet quality, elevated fasting serum insulin levels and increased risk of T2DM over approximately 8 years of follow‐up (Sarin et al. 2019). Unlike the population‐based longitudinal study by Sarin et al., the present study was conducted in a clinical sample of individuals already diagnosed with T2DM. Therefore, our findings may extend previous evidence by suggesting that food neophobia is not only associated with metabolic risk and diet quality in the general population, but may also be relevant to dietary diversity and treatment adherence among individuals with established T2DM. In this study, it was found that the treatment adherence scale score of food neophobic individuals (X̄ = 111.8 ± 30.07) was significantly higher than that of neophilic (X̄ = 59.5 ± 22.20) and neutral (X̄ = 83.0 ± 7.86) individuals (*p* < 0.05) (Table 6). These results indicate that higher food neophobia scores were associated with poorer treatment adherence among individuals with T2DM. Psychological barriers related to food neophobia may be linked to lower motivation for treatment and greater difficulty in adapting to the lifestyle changes required during the treatment process.

Studies have shown that there is a relationship between food neophobia and food choice and dietary diversity; studies have shown that dietary diversity decreases in individuals with food neophobia (Costa et al. 2020; Hopkins et al. 2023; Michel et al. 2021). It has also been reported that higher scores on food neophobia are associated with lower intakes of vitamin C, magnesium, fruit and vegetables, and higher intakes of simple sugars (Hazley et al. 2022). These findings are consistent with the present study, which showed that higher food neophobia was related to lower dietary diversity. However, while Hazley et al. focused on nutrient intake and food group consumption, the present study additionally examined treatment adherence, which is particularly important in the management of T2DM. In a study by Capiola and Raudenbush (Capiola and Raudenbush 2012), neophobic individuals were found to have diets with less food variety and lower total energy intake compared to neophilic individuals. In their study on food neophobia and dietary diversity, Ucar and Kizil (2018) found that higher FNS scores were associated with lower nutritional quality according to the Healthy Eating Index. In a study assessing the relationship between the level of food neophobia in adults and their eating habits and frequency of food consumption, Çakır et al. (2023) found that meat, egg, and legume group foods were consumed in higher amounts in neophilic individuals (176.14 ± 96.58 g/day) compared to neutrals (160.30 ± 96.48 g/day), and fresh fruit consumption was higher in neophobic individuals (137.38 ± 124.94 g/day) compared to neutrals (107.51 ± 101.57 g/day) (Çakır et al. 2023). Another study reported that greater food neophobia was linked to lower preference for vegetables, fruits, fish, desserts, and carbohydrate‐based foods (Catamo et al. 2024). Research from Finland also indicated that food neophobia among adults aged 19–45 years was related to reduced vegetable intake (women: 15 vs. 10 servings/week; men: 13 vs. 7 servings/week) and lower overall diet quality, and may create difficulties in following dietary recommendations (Knaapila et al. 2015). Taken together, these studies from different populations generally support the view that food neophobia is associated with less favorable dietary behaviors and lower dietary diversity. However, differences in age, cultural dietary patterns, health status, and study design should be considered when comparing these findings with the present study. According to the results of this study, a significant association was found between DDS and FNS for both groups (*p* < 0.001). It was observed that the dietary diversity score of individuals with T2DM and food neophobia who participated in this study was significantly lower than that of neophilic and neutral individuals (p < 0.001) (Table 5). Food neophobia may be associated with lower dietary diversity by causing individuals to reject new and different foods, making it difficult to follow the adequate and balanced diet recommended during the treatment process.

Food neophobia may also be relevant in other chronic diseases requiring long‐term dietary management. Zysk et al. (2019) reported higher food neophobia scores among individuals with celiac disease following a gluten‐free diet compared with individuals following a gluten‐free diet by their own choice. Although celiac disease and T2DM require different dietary approaches, both conditions involve long‐term dietary regulation. This comparison supports the view that food‐related avoidance behaviors may be relevant to adherence to dietary recommendations in chronic disease management.

Quick et al. (2014) found that neophobia and selectivity were negatively correlated with dietary diversity. In addition, neophobia and selectivity were individually negatively associated with diabetes management adherence. Conversely, dietary diversity showed a positive relationship with diabetes management compliance (Quick et al. 2014). Although Quick et al. conducted their study among youth with type 1 diabetes, whereas the present study included adults with T2DM, both studies indicate that food‐related behavioral traits may be associated with dietary diversity and adherence‐related behaviors in diabetes management. When food neophobia, dietary diversity, and treatment adherence scale scores of individuals with T2DM in this study were evaluated, a significant positive correlation was found between food neophobia score and treatment adherence scale score (*p* < 0.001). A high treatment adherence scale score means that the individual is not adhering to treatment. In this study, individuals with higher food neophobia scores tended to have lower treatment adherence among people with type 2 diabetes. A significant inverse relationship was found between the food neophobia scores and dietary diversity scores (*p* < 0.05) (Table 7), indicating that higher food neophobia was associated with decreased dietary diversity in individuals with T2DM. One possible explanation may be that some individuals with T2DM avoid unfamiliar foods because of concerns about their potential effects on blood glucose levels. These findings may indicate that individuals with higher levels of food neophobia are more likely to choose familiar foods, which may contribute to reduced‐dietary diversity and lower adherence to diabetes treatment.

The findings should be considered within the context of certain limitations. First, although this study was designed as a pilot study, the relatively small sample size, including 35 individuals with T2DM and 35 healthy controls, limits the statistical power of the analyses and restricts the generalizability of the findings. Second, due to the cross‐sectional design, causal interpretations cannot be made and the direction of the observed associations cannot be determined. Furthermore, as the study population was recruited by convenience sampling from a single center, the findings may not be representative of all individuals with T2DM. Accordingly, further studies with larger sample sizes, multicenter designs, and longitudinal follow‐up are needed to confirm these findings and better determine the direction of the observed relationships. Despite these limitations, this study is, to our knowledge, this study is the first to investigate the relationship between food neophobia, dietary diversity, and adherence to T2DM treatment in individuals with T2DM and to compare these parameters with a healthy control group.

## 5 Conclusions

Our findings indicate that higher food neophobia scores were associated with lower dietary diversity and poorer adherence to T2DM treatment among individuals with T2DM. Medical nutrition therapy is essential for glycemic control in people with diabetes, and dietary diversity is an important component of effective dietary management. Food neophobia may be associated with less favorable dietary behaviors in individuals with T2DM, which may be reflected in lower dietary diversity and potentially poorer nutrient adequacy. In addition, higher food neophobia may be linked to poorer treatment adherence, which may be relevant for diabetes management. Dietitians may have an important role in identifying and addressing food neophobia, improving dietary habits and encouraging healthy food choices. Further research with larger sample size, multicenter design, and longitudinal follow‐up is needed to validate the current results, clarify the direction of the associations, and evaluate their generalizability across different populations.

## Author Contributions

**Merve Sena Topkaya:** conceptualization, project administration, methodology, formal analysis, visualization, resources, writing – original draft, writing – review and editing. **Dilara Berşan Konyalıgil:** resources, project administration, formal analysis, writing – review and editing, visualization, methodology, writing – original draft, conceptualization. **Berna Betül Cihan:** writing – review and editing, investigation, conceptualization, writing – original draft, resources, project administration. **Kübra Tel Adıgüzel:** writing – review and editing, project administration, supervision. **Ertuğrul Ekici:** investigation. **Nevra Koç:** writing – review and editing, supervision, project administration.

## Funding

The authors have nothing to report.

## Disclosure

Author approval: All authors have read and approved the final version of the manuscript. Corresponding Author Kübra Tel Adıgüzel had full access to all of the data in this study and takes complete responsibility for the integrity of the data and the accuracy of the data analysis.

## Ethics Statement

The study was conducted according to the guidelines of the Declaration of Helsinki, and approved by the Ethics Committee of Gülhane Health Sciences University (decision no: 2024‐245 and date of approval: 28.05.2024).

## Consent

Informed consent was obtained from all subjects involved in the study.

## Conflicts of Interest

The authors declare no conflicts of interest.

## Data Availability

The data that support the findings of this study are available from the corresponding author upon reasonable request.
